# Bioassay-Guided Isolation and Antioxidant Evaluation of Flavonoid Compound from Aerial Parts of *Lippia nodiflora* L.

**DOI:** 10.1155/2014/549836

**Published:** 2014-05-25

**Authors:** A. Sudha, P. Srinivasan

**Affiliations:** Department of Bioinformatics, Alagappa University, Karaikudi, Tamil Nadu 630004, India

## Abstract

The present study was designed to identify the DPPH (2, 2-diphenyl-1-picrylhydrazyl) free-radical scavenging constituents from methanol extract of *L. nodiflora* using bioassay-guided fractionation. The ethyl acetate fraction (EAF) revealed a strong antioxidant activity, compared to other fractions through *in vitro* DPPH radical-scavenging assay. The repeated fractionation of active EAF by silica gel column chromatography yielded a compound with strong antioxidant potential. The isolated bioactive compound was determined as 2-(3, 4-dimethoxyphenyl)-5-hydroxy-7-methoxy-4H-chromen-4-one (5-hydroxy-3′, 4′, 7-trimethoxyflavone), by comparing spectral data (UV, IR, ^1^H NMR, ^13^C NMR, and MS) with literature reports. The isolated compound demonstrated an excellent antioxidant activity through all antioxidant assays and also significantly inhibited lipid peroxidation at a concentration of 50 **μ**g/mL. The results obtained suggested that extracts from *L. nodiflora* or its derived phytocompound can be used potentially as a bioactive source of natural antioxidants by contributing beneficial health effects.

## 1. Introduction

Reactive oxygen species (ROS) are continuously formed as a by-product of metabolisms in aerobic organisms and are also produced on exposure to tobacco smoke, ozone, radiations, organic solvents, and other environmental pollutants [1]. ROS play an important role in various physiological processes, including energy production, phagocytosis, cellular signal transduction, cell proliferation, differentiation, and apoptosis. On the other hand, increasing evidence highlights that overproduction of ROS can induce oxidative damage to all biomolecules (lipids, carbohydrates, proteins, enzymes, DNA, and RNA) and acts as a mediator of numerous disorders, for example, inflammation, arthritis, diabetes, arteriosclerosis, cancer, genotoxicity, and neurological disorders such as Alzheimer's disease [2]. Antioxidants are very essential for averting degenerative reactions produced by free radicals and reactive oxygen species, which have been concerned with many diseases and in food deterioration and spoilage [3]. However, the safety of some of the synthetic antioxidants used in the food industry has been questioned, because recent studies recognized that they might be carcinogenic [4]. Hence, there is an emerging interest in natural antioxidants, which might help to prevent oxidative damage [5].

Plants and plant products are magnificent sources of phytochemicals and have been found to hold an array of biological activities including antioxidant potential [6]. Plants synthesize antioxidant compounds, mostly flavonoids and polyphenols, which have been reported to protect the human body from various diseases by neutralizing ROS. Recently, phenolic compounds have received increasing significance among various phytochemicals, due to their wide distribution in the plant kingdom and for their biological activities, namely, anticarcinogenic, antiatherogenic, anti-inflammatory, antimicrobial, and antioxidant activities [7–9]. Antioxidants can be either used as dietary, food supplement or as a drug by the humans [10]. Several studies have revealed that the increased dietary intake of natural antioxidants, such as flavonoids and other phenolic compounds, almost present in plants, exhibits potential protective effects against many degenerative diseases [11–13].

*Lippia nodiflora* Linn. (Verbenaceae), commonly called Poduthalai in Tamil, is a creeping perennial herb and locally abundant in wet regions, and several medicinal properties are attributed to this plant in the traditional system of medicine. The infusion of the leaves and tender stalks is given to children suffering from indigestion and to women after delivery. The chutney made from the leaves and fruits are often taken to relieve the irritation of internal piles [14]. Numerous pharmacological properties of* L. nodiflora* including anti-inflammatory, antipyretic, antitussive, antidiabetic, and antimelanogenesis effects have been reported [15–18]. The ethnopharmacological relevance of* L*.* nodiflora* for skin diseases and in folk cosmetics, such as pimples, carbuncle, and skin burns, has also been revealed [19]. The phytochemical constituents of* L. nodiflora*, such as flavonoids [20, 21], flavone glycosides [22], alkaloids, essential oils, resin [23], quinol [24], and steroids [25, 26], have been previously reported. Hence, these phytochemicals are considered to be accountable for the pharmacological properties of this plant. Even though there is an evidence for the antioxidant activity of methanolic extract of* L. nodiflora*, the major antioxidative constituents present in the aerial parts have not been extensively investigated. Hence, the present study was undertaken to isolate active compounds responsible for the antioxidant property of methanol extract of* Lippia nodiflora* L. through bioassay-guided fractionation using* in vitro* DPPH assay.

## 2. Materials and Methods

### 2.1. General

Nuclear magnetic resonance spectra were recorded on BRUKER, Avance 400 MHz (Switzerland) NMR instrument, operating at 400 MHz for ^1^H and 100 MHz for ^13^C nuclei at room temperature and referenced to the residual solvent signal. The chemical shift and coupling constants (*J*) values are reported in ppm and Hz, respectively. HPLC analysis was performed using a C-18 column (250 × 4.6 mm, 5 *μ*) in a Shimadzu LC-8A chromatographic apparatus (Shimadzu, Singapore). The mobile phase consisted of methanol-0.5% phosphoric acid in water (60 : 40, v/v) and the flow rate was held constant at 1 mL/min. The peaks are detected at 280 nm, using variable wavelength UV detector. Silica gel 60 F_254_ plates (20 × 20 cm, 0.2 mm thick; E-Merck, Germany) were used for thin-layer chromatography (TLC) analysis. The ultraviolet spectra were recorded using Varian Cary 500 scan spectrophotometer,  *λ*_max⁡_  (log⁡*ε*) in nm, whereas the FTIR spectrum was obtained using a Nicolet 380 (Thermo Scientific, USA). The functional group was identified using potassium bromide (KBr) and scanned in the range of 4000–400 cm^−1^. Sample was dissolved in methanol and ESI mass spectra were obtained with a Thermo Scientific Exactive Mass Spectrometer (Thermo Fisher Scientific, USA).

The reagents such as 2-deoxy-D-ribose, butylated hydroxyl toluene (BHT), 2, 2-diphenyl-1-picrylhydrazyl (DPPH), phenazine methosulphate (PMS), nitroblue tetrazolium (NBT), sodium nitroprusside, and Griess reagent were obtained from (Sigma Chemicals St. Louis, MO, USA). 2, 4, 6-Tripyridyl-S-triazine (TPTZ), thiobarbituric acid (TBA), trichloroacetic acid (TCA) ethylene diaminetetraacetic acid (EDTA), ferric chloride (FeCl_3_), hydrogen peroxide (H_2_O_2_), and nicotinamide adenine dinucleotide-reduced (NADH) were obtained from M/s (Sisco Research laboratories, Mumbai, India). HPLC grade solvents and reagents used for extraction and silica gel (0.075–0.15 mm) for column chromatography were obtained from M/s (Sisco Research laboratories, Mumbai, India). All other chemicals and reagents used in this study were of analytical grade.

### 2.2. Plant Materials

The plant materials were freshly collected between August and September 2011, from Karaikudi, Sivagangai district, Tamil Nadu. The plant was taxonomically identified and authenticated by Dr. G. V. S. Murthy, Joint Director, Botanical Survey of India, Tamil Nadu Agricultural University Campus, Coimbatore (BSI/SRC/5/23/2012-13/Tech-19). A voucher specimen of the plant* L. nodiflora* (DBI-HP: Specimen-1) was prepared and preserved in the Molecular Biology Lab, Department of Bioinformatics, Alagappa University, Karaikudi, Tamil Nadu.

### 2.3. Extraction, Fractionation, and Isolation of Antioxidant Compound

The aerial parts (stem, leaves, and flowers) were washed with tap water, shade-dried, and reduced to fine powder. The powdered aerial parts of* L. nodiflora* (1.5 kg) were extracted with 90% methanol (4.5 L × 2) at room temperature. The mixture was filtered through Whatman number l filter paper and the solvents from the combined extract were concentrated using a vacuum rotary evaporator (Superfit, India), at 60°C to afford 64.7 g of crude methanol extract (4.31%). The solvent was selected based on its yield from preliminary extraction and phytochemical screening studies. The extract was dissolved in 500 mL of warm water, and the resulting aqueous portion was partitioned with ethyl acetate (EtOAc) (4 × 200 mL) using a separating funnel to afford ethyl acetate fraction (EAF) and aqueous portion. The aqueous phase was then successively partitioned with n-butanol (3 × 200 mL), thus obtaining n-butanol soluble fraction (BF) and water fraction (WF). All the fractions were collected separately and reduced using a vacuum rotary evaporator to remove the solvent and the resultant aqueous extract was lyophilized in vacuo. The samples were then tested for its antioxidant property using 2, 2-diphenyl-1-picrylhydrazyl (DPPH^•^) assay.

The DPPH^•^ active EAF (17.2 g) was loaded as a dried slurry of silica gel to column chromatography (45 × 3.5 cm) and eluted with petroleum ether: EtOAc gradient elution (100 : 0—0 : 100), in increasing order of polarity. A total of 92 fractions of 100 mL each were collected and analyzed by TLC (Silica gel F_254_ plates 20 × 20 cm, Merck, Germany). TLC analysis was carried out using ethyl acetate: chloroform: formic acid (5 : 4 : 1) as the mobile phase and the separated bands were visualized using iodine vapors and vanillin-sulphuric acid reagent. These fractions were pooled to afford seven major fractions (Fr. A: 1–13, Fr. B: 14–35, Fr. C: 36–48, Fr. D: 49–60, Fr. E: 61–70, Fr. F: 71–80, and Fr. G: 81–92) based on TLC analysis. These fractions were tested for bioactivity using DPPH spectrophotometric assay. For further purification, the highly active Fr. B (2.8 g) was loaded on a silica gel column, eluted with petroleum ether-EtOAc gradients, and the ethyl acetate content of the mixture was increased in a series of 5% steps. The inactive and less active proved fractions were discarded. Finally, the active Fr. B_2_ eluted with petroleum ether-ethyl acetate (85 : 15) yielded 117 mg of compound. The purity of isolated compound was established by HPLC and its structure was confirmed through the interpretation of the spectral data (UV, FT-IR, ^1^H, ^13^C NMR, and MS) and further tested for its antioxidant effects.

### 2.4. Antioxidant Activities

#### 2.4.1. DPPH Radical Scavenging Assay

The DPPH radical scavenging activities of methanol extract, EAF, BF, and WF were tested according to Yamaguchi et al. [27]. Briefly, 0.2 mL of the sample solutions of different concentrations was added to 1 mL of 0.1 mM of freshly prepared DPPH solution. The reaction mixtures were shaken vigorously and absorbance at 517 nm was determined after 20 min at room temperature. Control sample was prepared containing the same volume without test compounds or reference antioxidants, while DMSO was used as blank. The reference antioxidant BHT was used as the positive control in all the assays. The radical scavenging activity was measured as a decrease in the absorbance of DPPH^•^ and calculated as follows:

(1) $$\begin{array}{c} \text{Scavenging effect (\%)}=\left[\frac{A_{\text{control}}-A_{\text{sample}}}{A_{\text{control}}}\right]\times 100, \end{array}$$

where *A*_control_ is the absorbance of the control and *A*_sample_ is the absorbance of the extract or fractions or standard.

#### 2.4.2. Superoxide Radical-Scavenging Assay

The superoxide radical-scavenging effect was determined by the method of Nishikimi et al. [28]. The reaction mixture with NBT (1 mM) in phosphate buffer (0.1 M, pH 7.4), NADH (1 mM) with or without samples, and PMS (0.1 mM) was incubated at room temperature for 5 min and the absorbance was recorded at 560 nm. The inhibition percentage was calculated against a control without the samples. The scavenging ability was calculated using the equation as described for DPPH assay.

#### 2.4.3. Hydroxyl Radical Scavenging Assay

The capacity of the extract and compound to reduce hydroxyl radical-mediated peroxidation was carried out by the method of Hinneburg et al. [29]. Briefly, 0.5 mL of 5.6 mM 2-deoxy-D-ribose in KH_2_PO_4_−NaOH buffer (50 mM, pH 7.4), 0.2 mL of 100 *μ*M FeCl_3_, and 104 mM EDTA (1 : 1  v/v) solution were added to 0.1 mL of different concentrations of test samples, followed by 100 *μ*L of 1.0 mM H_2_O_2_ and 0.1 mL of 1.0 mM aqueous BHT. The reaction mixtures were shaken vigorously and incubated at 50°C for 30 min. Subsequently, 1 mL of 2.8% TCA and 1 mL of 1.0% TBA were added to each tube containing reaction mixture and samples were mixed well again and boiled in a water bath at 50°C for 30 min. The absorbance of solution was read at 532 nm. The hydroxyl radical scavenging ability was calculated using the formula as described for DPPH assay and the values are presented as means of triplicate analyses.

#### 2.4.4. FRAP (Ferric Reducing Antioxidant Power) Assay

The FRAP assay was determined by the method of Benzie and Strain [30] with minor modifications. It depends on the ability of the sample to reduce the ferric tripyridyltriazine (Fe (III)-TPTZ) complex to ferrous tripyridyltriazine (Fe (II)-TPTZ) at low pH. Fe (II)-TPTZ has an intensive blue color which can be read at 593 nm. The stock solutions consist of 300 mM acetate buffer (pH 3.6), 10 mM 2, 4, 6 tripyridyl S triazine (TPTZ) in 40 mM of HCl, and 20 mM ferric chloride solution. The fresh working solution was prepared by mixing 25 mL of acetate buffer, 2.5 mL of TPTZ, and 2.5 mL of FeCl_3_·6H_2_O and the temperature was maintained to 37°C before use. The various concentrations of extract, compound, and BHT (10–50 *μ*g/mL) were allowed to react with 2 mL of the FRAP solution for 30 min in the dark condition. The absorbance was recorded at 593 nm. The results are expressed in *μ*M Fe (II)/g and were estimated using aqueous FeSO_4_·7H_2_O (20–100 *μ*M) as standard for calibration.

#### 2.4.5. Nitric Oxide Radical Scavenging Assay

At physiological pH, nitric oxide generated from sodium nitroprusside in aqueous solution interacts with oxygen to produce nitrite ions, which were measured by the Griess reaction [31]. Briefly, 3 mL of the reaction mixture containing 10 mM sodium nitroprusside and the test samples (10–50 *μ*g/mL) in phosphate buffer were incubated for 150 min at 25°C. After incubation, 0.5 mL of the reaction mixture was mixed with 1 mL of sulfanilic acid reagent (0.33% in 20% glacial acetic acid) and allowed to stand for 5 min for complete diazotization. Then, 1 mL of naphthyl ethylene diamine dihydrochloride (0.1% w/v) was added and the mixture was allowed to stand for 30 min at 25°C. A pink colored chromophore is generated and the absorbance was measured spectrophotometrically at 540 nm against a blank sample. The nitric oxide radical scavenging activity of the extract and compound is reported as % inhibition and was calculated using the formula as described for DPPH assay.

#### 2.4.6. Lipid Peroxidation Assay

Lipid peroxidation (LPO) assay was performed according to the protocol described by Damien et al. [32] to measure the lipid peroxide formed, using egg yolk homogenates as lipid-rich media. Briefly, 0.5 mL of egg homogenate (10% v/v prepared in 1.15% w/v KCl) was added to 0.1 mL of each test samples (10–50 *μ*g/mL) taken in a test tube and made up to 1 mL with double distilled water. Thereafter, 0.05 mL of FeSO_4_ (0.07 M) was added to induce lipid peroxidation, and the mixture was incubated for 30 min at room temperature. Then, 1.5 mL of 3.5 M acetic acid (pH adjusted to 3.5) was added, followed by 1.5 mL of TBA (0.06 M) in sodium dodecyl sulphate (0.04 M). The resulting mixture was vortex and heated at 95°C for 1 hr. After cooling, 5 mL of butanol was added to each tube and centrifuged at 3000 rpm for 10 min. The absorbance of the organic upper layer was measured at 532 nm and the above procedure was followed for the control by using 0.1 mL of SDS instead of the test sample. The percentage inhibition was calculated according to the following formula:

(2) Percentage inhibition of lipid peroxidation=(1−EC)×100,

where *E* is the absorbance of the test sample and *C* is the absorbance of the fully oxidized control.

### 2.5. Statistical Analysis

The experiments were carried out in triplicate and data were expressed as means ± standard deviation (SD). All statistical analyses were performed using graph pad prism (version 5.0; Graph Pad software Inc. San Diego, CA, USA). The IC_50_ value represented the concentration of the test samples that caused 50% inhibition. *P* values <0.05 were considered as significant.

## 3. Results and Discussion

### 3.1. Isolation and Structure Determination of Antioxidant Compound

**Figure 1:**
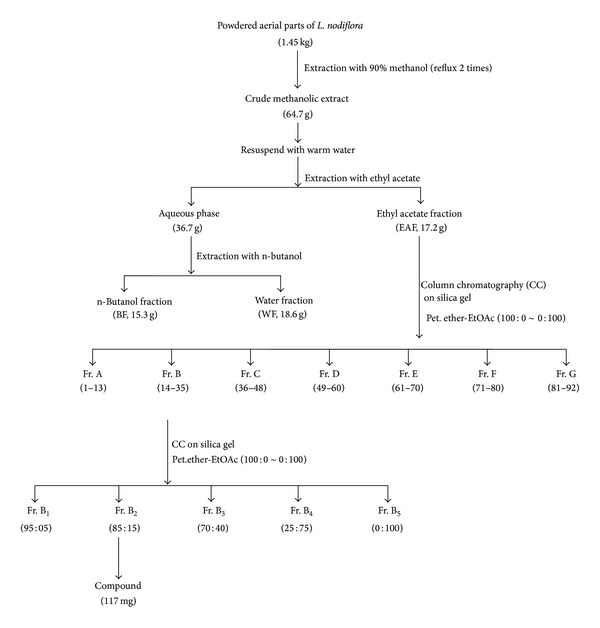
Extraction scheme for the isolation of antioxidant compound from aerial parts of* Lippia nodiflora* L.

**Figure 2:**
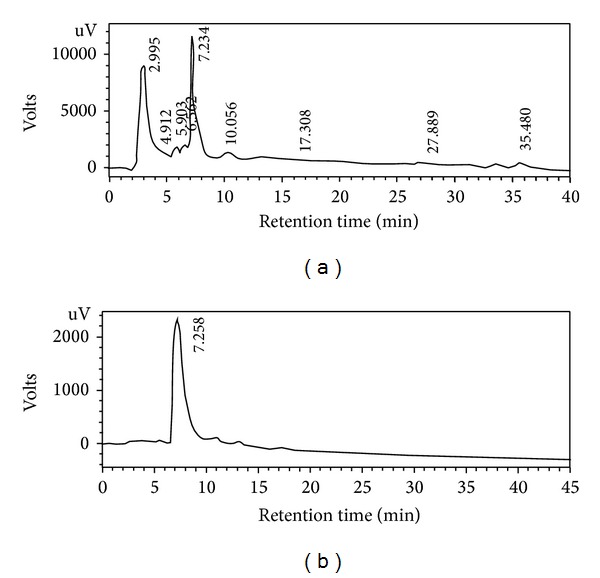
(a) HPLC chromatogram of whole methanol extract of* L. nodiflora*. (b) HPLC chromatogram of isolated compound (5-hydroxy-3′, 4′, 7-trimethoxyflavone) at 280 nm. The HPLC profile shows the purity of the isolated compound from aerial parts of* L. nodiflora*.

The methanol extract and potential antioxidant fractions of* L. nodiflora* were initially screened by spectrophotometric DPPH assay and the results are shown in Table 1. The DPPH assay revealed that the methanol extract had significant scavenging effects with increasing concentrations in the range of 10–50 *μ*g/mL. Moreover, the scavenging effect of methanol extract was significantly similar to that of standard BHT. At 50 *μ*g/mL, methanol extract and BHT exhibited 79.35% and 86.42% inhibition and the IC_50_ values (the concentration with scavenging activity of 50%) were 24 and 19 *μ*g/mL, respectively. The result obtained herein was lower than the reported scavenging activity of methanol extract of* L. nodiflora* (12.03 *μ*g/mL) [33]. Among the fractions, the DPPH^•^ was significantly scavenged by ethyl acetate fraction (EAF) in a dose-dependent manner, with an IC_50_ value of 26 *μ*g/mL, followed by WF and BF, with IC_50_ values of 66 and 83 *μ*g/mL (Table 1). Hence, the EAF was subjected to repeated bioassay-guided fractionation on silica gel column chromatography using petroleum ether: EtOAc gradient elution system. The further purification of active fractions obtained from silica gel column chromatography finally yielded a bioactive antioxidant compound from the petroleum ether-ethyl acetate (85 : 15 v/v) mixture. The extraction procedure for the isolation of active compound was schematically shown in Figure 1.  Figure 2(a) shows the HPLC profile of the chemical constituents present in the 90% methanol extract of* L. nodiflora*. The HPLC chromatogram of purified compound exposed the presence of a peak, with a retention time of 7.2 min, eluted isocratically with the mobile phase of methanol-0.5% phosphoric acid in water (60 : 40, v/v) (Figure 2(b)).

**Figure 3:**
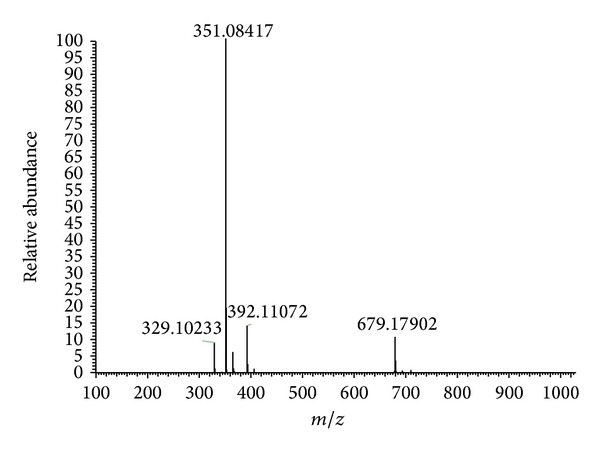
Molecular mass of isolated compoundfrom aerial parts of* L. nodiflora*.

**Figure 4:**
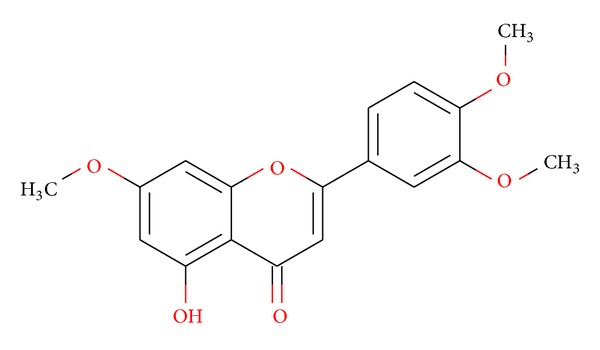
Structure of isolated compound (5-hydroxy-3′, 4′, 7-trimethoxyflavone) from ethyl acetate (EAF) fraction of aerial parts of* L. nodiflora*.

The compound was obtained as pale white amorphous powder (yield: 117 mg, 0.68%). The FTIR peaks determined the bonds relevant to alcoholic O–H stretching (3267 cm^−1^), =C–H stretching (3010 cm^−1^), –C–H stretching (2975 cm^−1^), –C=O stretching (1605 cm^−1^), –C=C stretching (1506 cm^−1^), –C–H bending (1154 cm^−1^), C–O stretching (1315 cm^−1^), =C–H bending (1030 cm^−1^), and O–H bending (993 cm^−1^). ^1^H NMR (CDCl_3_, 400 MHz): *δ* (ppm) 3.91 (3H, *s*, 4′-OMe), 3.89 (3H, *s*, 7-OMe), 3.87 (3H, *s*, 3′-OMe), 6.49 (*s*, 1H, OH), 6.64 (*d*, *J* = 6.63 Hz,  1H, H6), 6.898 (*s*, 1H, H8), 6.99 (*d*, *J* = 6.3 Hz, 1H, H2′), 7.52 (*d*, *J* = 6.3 Hz,  1H, H5′), 8.178 (s, 1H, H6′); ^13^C NMR (CDCl3, 100 MHz): *δ* (ppm) 171.61 (C-4), 144.30 (C-4′), 125.69 (C-1′), 162.87 (C-2), 161.77 (C-3′) 156.82 (C-5), 130.87 (C-6′), 114.28 (C-5′), 137.29 (C-9), 103.92 (C-2′), 157.74 (C-7), 98.92 (C-6), 98.19 (C-8), 111.47 (C-3), 113.58 (C-10), 54.51 (7-OMe), 54.76 (3′-OMe), 54.88 (4′-OMe) (Figures S_1_ and S_2_ of the Supplementary Information available online at http://dx.doi.org/10.1155/2014/549836). The compound exhibits a molecular weight of 329 (ESI-MS; *m*/*z* 351.084 [M + Na]^+^) with an elemental formula of C_18_H_16_O_6_ (Figure 3). From these spectral interpretations, the isolated compound has been characterized as 2-(3, 4-dimethoxyphenyl)-5-hydroxy-7-methoxy-4H-chromen-4-one (5-hydroxy-3′, 4′, 7-trimethoxyflavone) (Figure 4), which agreed with the data reported [34].

### 3.2. Antioxidant Activities of Methanol Extract and Isolated Compound from Ethyl Acetate Fraction (EAF)

Flavonoids, a major group of polyphenols, are considered to be the active principles in diverse medicinal plants and have been reported to possess numerous pharmacological properties. The most essential biological activity of flavonoids is mainly due to their antioxidant property by acting as radical scavengers, hydrogen donors, reducing agents, and peroxidation inhibitors [35]. Previous studies reported that the pharmacological effects of* L. nodiflora*, such as antioxidant, diuretic, anti-inflammatory, and antimicrobial activities, were recognized due to the presence of the phenol and flavonoid compounds [33, 36]. In the present study, the isolated compound was identified as flavone and its antioxidant activities were examined by using various* in vitro* antioxidant models.

#### 3.2.1. DPPH Radical Scavenging Effect

**Figure 5:**
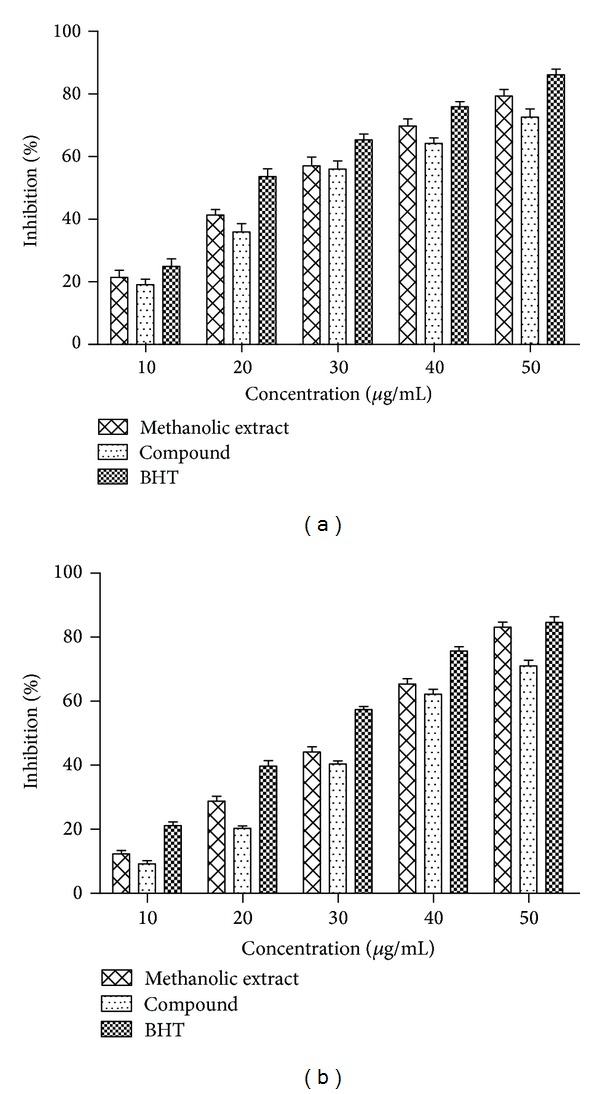
(a) DPPH radical scavenging and (b) superoxide radical scavenging activities of extract, compound, and BHT at different concentrations. Values are mean ± SD (*n* = 3).

The substances are considered to be antioxidants, when they are capable of reducing the stable DPPH radical (purple) to the nonradical form DPPH-H (yellow), and thus they act as radical scavengers due to their hydrogen donating abilities [37]. The results of DPPH scavenging activity of all test samples are presented in Figure 5(a). The scavenging activity of methanolic extract, compound, and BHT increased with an increase in sample concentration (10–50 *μ*g/mL). The highest DPPH scavenging activity for extract, compound, and BHT was found to be 79.35%, 72.66%, and 86.09%, respectively, at 50 *μ*g/mL. It should be noted that the scavenging activity of compoundwas found to be close to the extract. The IC_50_ values of scavenging activity on DPPH radical of extract and BHT are given in Table 1, whereas, for compound, it is found to be 27 *μ*g/mL. From these obtained data, the extract and compound were considered as an effective free-radical inhibitor as well as the primary antioxidants, which may limit free-radical damage that takes place in the body.

#### 3.2.2. Superoxide Radical Scavenging Effect

The formation of reactive oxygen species such as hydroxyl radical, hydrogen peroxide, and singlet oxygen in living system was mainly due to the participation of superoxide anion radicals, either directly or widely through enzyme or metal catalyzed progression [38]. It was therefore anticipated to evaluate the relative interceptive capacity of the extract and compound to scavenge the superoxide radical. From the data presented in Figure 5(b), it was noted that the extract, compound, and BHT showed the highest radical scavenging activities (71.07%–84.64%) at 50 *μ*g/mL and the scavenging activity increased with increasing concentration of the samples. The scavenging ability of compound on superoxide radicals was found to be moderate compared to methanolic extract. However, the scavenging activities of extract (83.09%) were found to be very closer to that of BHT (84.64%), which is considered to be a strong superoxide radical scavenger. The IC_50_ value of plant extract, compound, and BHT was found to be 32, 38, and 26 *μ*g/mL, respectively.

#### 3.2.3. Hydroxyl Radical Scavenging Effect

**Figure 6:**
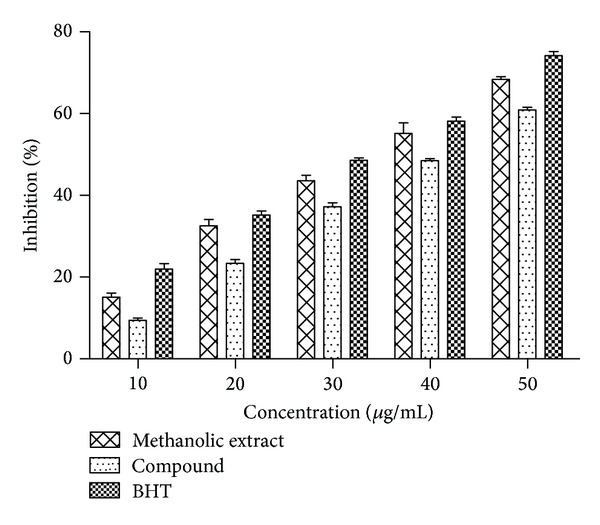
Hydroxyl radical scavenging effect of extract, compound, and BHT at different concentrations. Values are mean ± SD (*n* = 3).

Hydroxyl radical, an extremely known reactive oxygen species, was competent to attack and spoil almost every molecule in the living cells [11]. They were also capable of stimulating lipid peroxidation process rapidly by attacking the fatty acid side chains of the membrane phospholipids [38]. The scavenging activities of methanolic extract, compound, and BHT on hydroxyl radical inhibition are shown in Figure 6. All the examined samples showed significant hydroxyl radical scavenging activity at 50 *μ*g/mL concentration and the scavenging activity for extract, compound, and BHT was 68.38%, 60.90%, and 74.16%, respectively. The methanolic extract of* L. nodiflora* (IC_50_ = 36 *μ*g/mL) was more powerful than the compound (IC_50_ = 43 *μ*g/mL). The positive control, BHT, was extremely effectual on hydroxyl radical scavenging, with an IC_50_ value of 32 *μ*g/mL. This observed capacity of the extract and compounds to scavenge ^•^OH radical pointed out that the tested samples could considerably inhibit lipid peroxidation, since ^•^OH radicals are extremely distressed during peroxidation.

#### 3.2.4. FRAP (Ferric Reducing Antioxidant Power) Assay

The ferric reducing/antioxidant power (FRAP assay) is widely used in the assessment of the antioxidant component in dietary polyphenols [39]. The reducing properties are usually related to the presence of compounds which exert their action by breaking the free-radical chain by donating a hydrogen atom [40]. The results of reductive potential of plant extract and compound relative to BHT, a well-known antioxidant data, are shown in Table 2. The reducing ability of the extract was in the range of 23.46–71.14 *μ*M Fe (II)/g. The FRAP values for the methanol extract were significantly higher than that of compound and BHT, while the compound revealed the lowest FRAP values (11.18–51.70 *μ*M Fe (II)/g). At 50 *μ*g/mL, the FRAP value of* L. nodiflora* extract was found to be 71.14 compared to compound and BHT with FRAP value of 51.70 and 63.18, respectively (Table 2). This result implies that the isolated compound did not show reliable reducing power, when compared to its DPPH and superoxide radical scavenging abilities.

#### 3.2.5. Nitric Oxide Radical Scavenging Assay

**Figure 7:**
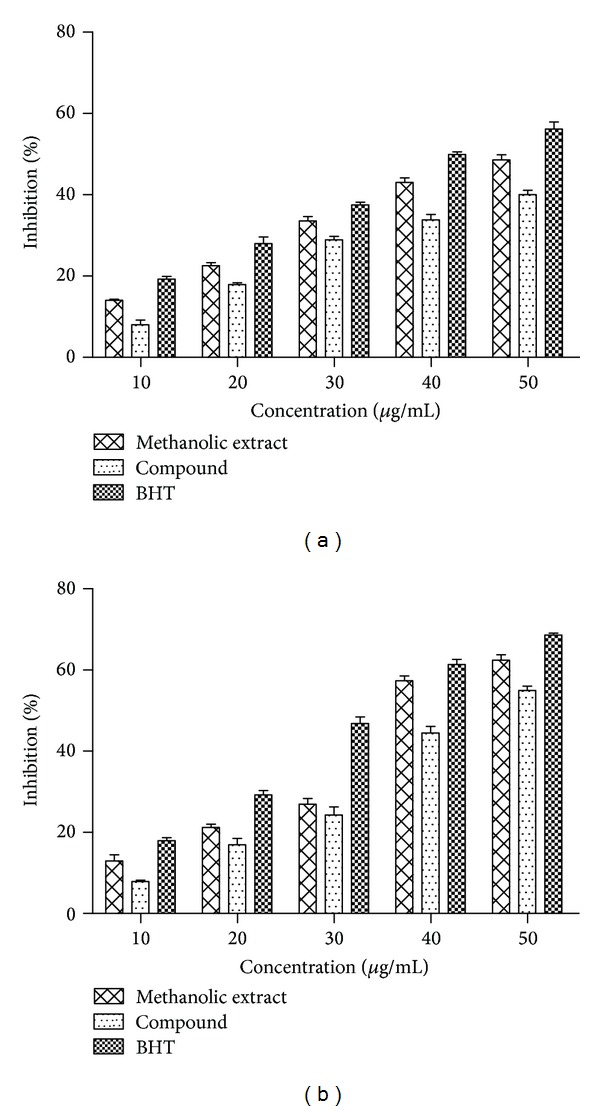
(a) Nitric oxide radical scavenging and (b) lipid peroxidation inhibition activities of extract, compound, and BHT at different concentrations. Values are mean ± SD (*n* = 3).

Nitric oxide radicals (NO^•^) play a vital role in inducing inflammatory response and their toxic effects increase only when they react with superoxide radicals that damage biomolecules like proteins, lipids, and nucleic acids [41]. The suppression of NO radicals release may be attributed to a direct (NO^•^) scavenging effect as both extract and compound decreased the amount of nitrite generated from the decomposition of sodium nitroprusside through* in vitro* studies as shown in Figure 7(a). The extract and compound at 50 *μ*g/mL exhibited 48.6% and 40% inhibition which was comparable to the standard BHT, which exhibited 49.8% inhibition at 40 *μ*g/mL. However, both extract (IC_50_ = 54 *μ*g/mL) and compound (IC_50_ = 67 *μ*g/mL) can inhibit NO radicals but lesser when compared to standard BHT (IC_50_ = 41 *μ*g/mL). The NO radical-scavenging activities of the extract and compound also followed a concentration-dependent pattern. The results suggest that the isolated flavone compound of* L. nodiflora* might be an effective and novel therapeutic agent for scavenging and regulating the pathological conditions caused by excessive generation of NO and its oxidation product, peroxynitrite.

#### 3.2.6. Inhibitory Activity towards Lipid Peroxidation

The disturbances in the membrane assembly lead to changes in fluidity and permeability, modifications of ion transport, and inhibition of metabolic processes, altogether revealed to be the collective consequence of reactive oxygen species formed during lipid peroxidation [42]. The extract and compound were capable of preventing MDA formation in a concentration-dependent manner (Figure 7(b)). At 50 *μ*g/mL, methanolic extract and compound possessed 62.44% and 54.95% inhibition, while BHT exhibited 68.64% inhibition at the same concentration. The IC_50_ values for extract and compound were found to be 39 and 46 *μ*g/mL. There is no significant difference in the IC_50_ values of extract and compound (*P* < 0.05). However, IC_50_ values of extract were in good agreement with BHT (IC_50_ = 34 *μ*g/mL). The presence of hydroxyl and electron-donating methoxy group in the compound (5-hydroxy-3′, 4′, 7-trimethoxyflavone) may be accountable for the antioxidant activity in all the experimental assays.

Based on* in vitro* antioxidant results of the present work, the methanol extract and isolated compound from ethyl acetate fraction of* L. nodiflora* were believed to be an electron donor, capable of counteracting free radicals. This is the first study to give an account on the antioxidant and free-radical scavenging activity of 5-hydroxy-3′, 4′, 7-trimethoxyflavone from* L. nodiflora*. The results of the present work also propose that the numerous pharmacological properties exerted by* L. nodiflora* may be partly due to the presence of antioxidant flavone compound.

## 4. Conclusion

The present study was projected to assess the antioxidant and free-radical scavenging activities of extract and fractions from aerial parts of* L. nodiflora* by using* in vitro* antioxidant models. The ethyl acetate fraction (EAF) exhibited highest free-radical scavenging activity, among the fractions. A bioassay-guided fractionation and purification of EAF resulted in the identification of the flavone compound, namely, 5-hydroxy-3′, 4′, 7-trimethoxyflavone. The measurement of antioxidant activity of the flavone compound, by using various* in vitro* antioxidant models, proved it to be a potent antioxidant compound. These results signify that methanol extract, ethyl acetate fractions, and isolated compound exhibited interesting antioxidant properties and afford an essential basis for the use of* L. nodiflora* in the treatment of oxidative damages. Furthermore, these findings hold great perception in the development of alternative antioxidant agents, and still further work is warranted to sort out and characterize the active principles from other fractions, in order to establish their therapeutic efficacy and mechanism of action.

## Supplementary Material


^1^H (400 MHz) and ^13^C NMR (100 MHz) spectra were recorded on a BRUKER, Avance 400 MHz NMR spectrometer, with tetramethylsilane (TMS) as an internal standard. The chemical shifts were given in ppm and coupling constants (J) in Hz. The complete proton and carbon assignments were based on 1D (^1^H, ^13^C) NMR experiments. The information from the ^13^C NMR spectrum displays 18 signals due to one carbonyl, three methoxy, six methine as well as eight quaternary carbon atoms which indicate the compound to be an aromatic one.

## Figures and Tables

**Table 1:** DPPH radical scavenging activity of the crude extract and fractions of aerial parts of *Lippia nodiflora* L.

Sample	DPPH (IC_50_) (*μ*g/mL)
CME	24.66 ± 1.1
EAF	26.06 ± 0.95
BF	83.62 ± 0.45
WF	66.39 ± 0.52
BHT^a^	19.12 ± 0.57

^a^BHT: Standard antioxidant; data represented as means ± SD (*n* = 3).

**Table 2:** Antioxidant potentials of extract, compound, and BHT at different concentrations examined by FRAP assay.

Sample	Test concentration (*μ*g/mL)	FRAP value (*μ*M Fe(II)/g)^a^
Methanol extract	10	23.4 ± 0.54
	20	27.5 ± 1.24
	30	52.5 ± 0.69
	40	63.9 ± 0.70
	50	71.1 ± 1.69

Compound	10	11.1 ± 1.00
	20	23.6 ± 0.57
	30	35.1 ± 0.63
	40	42.7 ± 0.58
	50	51.7 ± 0.83

BHT	10	20.7 ± 0.70
	20	30.0 ± 0.72
	30	48.5 ± 0.77
	40	54.7 ± 0.54
	50	63.1 ± 0.20

^a^The values are the average of three independent experiments.
