# Supplementary material for: Bioassay-Guided Isolation and Antioxidant Evaluation of Flavonoid Compound from Aerial Parts of Lippia nodiflora L
Source: Biomed Res Int. 2014 May 25;2014:549836. doi: 10.1155/2014/549836 (PMC4055635; doi:10.1155/2014/549836)
Supplement: Supplementary file 1 — 1H (400 MHz) and 13C NMR (100 MHz) spectra were recorded on a BRUKER, Avance 400 MHz NMR spectrometer, with tetramethylsilane (TMS) as an internal standard. The chemical shifts were given in ppm and coupling constants (J) in Hz. The complete proton and carbon assignments were based on 1D (1H, 13C) NMR experiments. The information from the 13C NMR spectrum displays 18 signals due to one carbonyl, three methoxy, six methine as well as eight quaternary carbon atoms which indicate the compound to be an aromatic one. [file 549836.f1.doc]

**Supplementary information**

**Bioassay-guided isolation and antioxidant evaluation of flavonoid compound from aerial parts of *Lippia nodiflora* L.**

1. **Sudha and P. Srinivasan***

Department of Bioinformatics, Alagappa University, Karaikudi -630004, Tamil Nadu, India

*** Corresponding Author**

**Dr. P. Srinivasan**

Assistant Professor

E-mail: sri.bioinformatics@gmail.com

**Supplementary legends**

**Figure S_1_:** Proton NMR profile of isolated compound from aerial parts of *L. nodiflora.*

**Figure S_2_:** Carbon NMR profile of isolated compound from aerial parts of *L. nodiflora.*


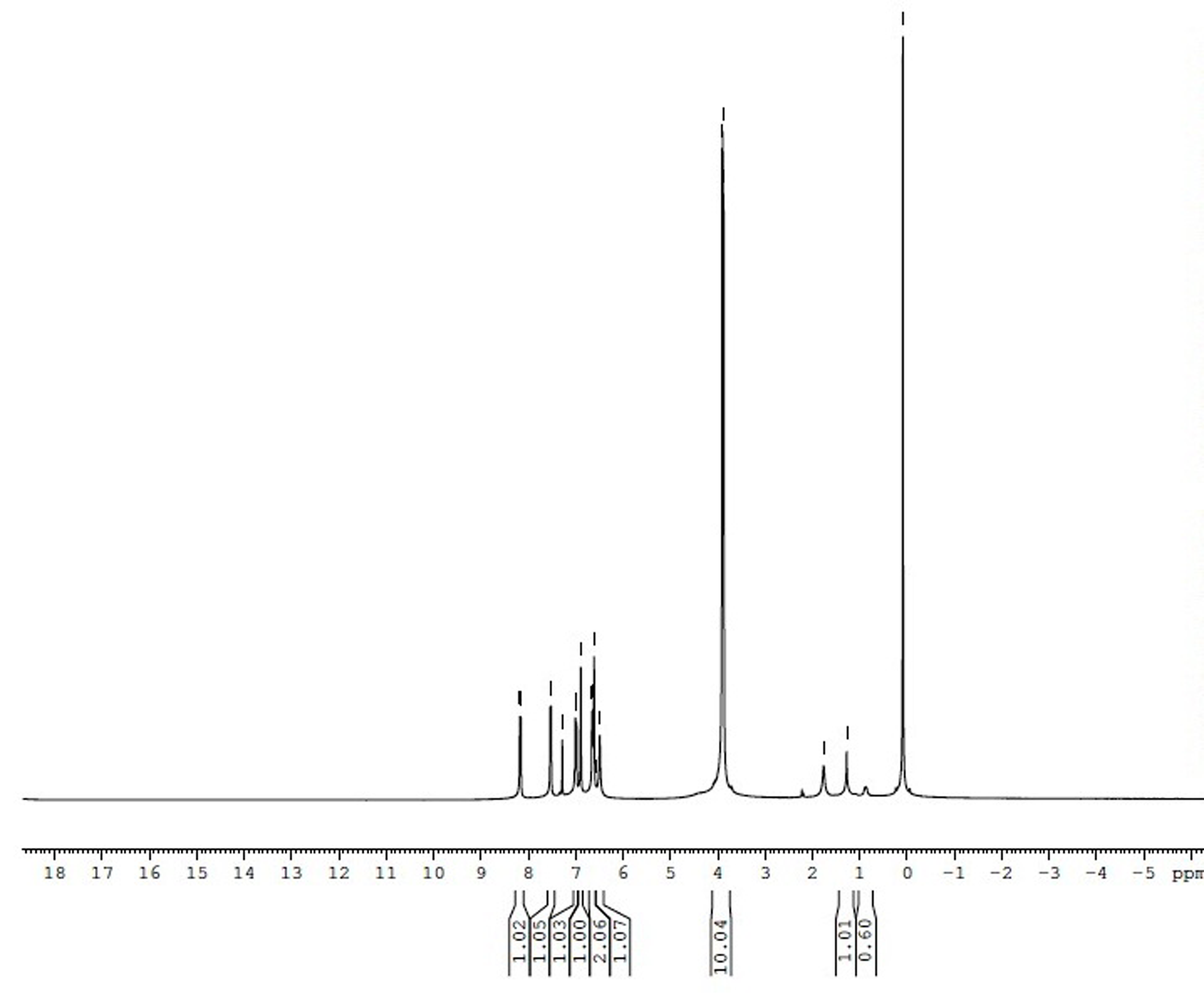


**Figure S_1_:** Proton NMR profile of isolated compound from aerial parts of *L. nodiflora.*


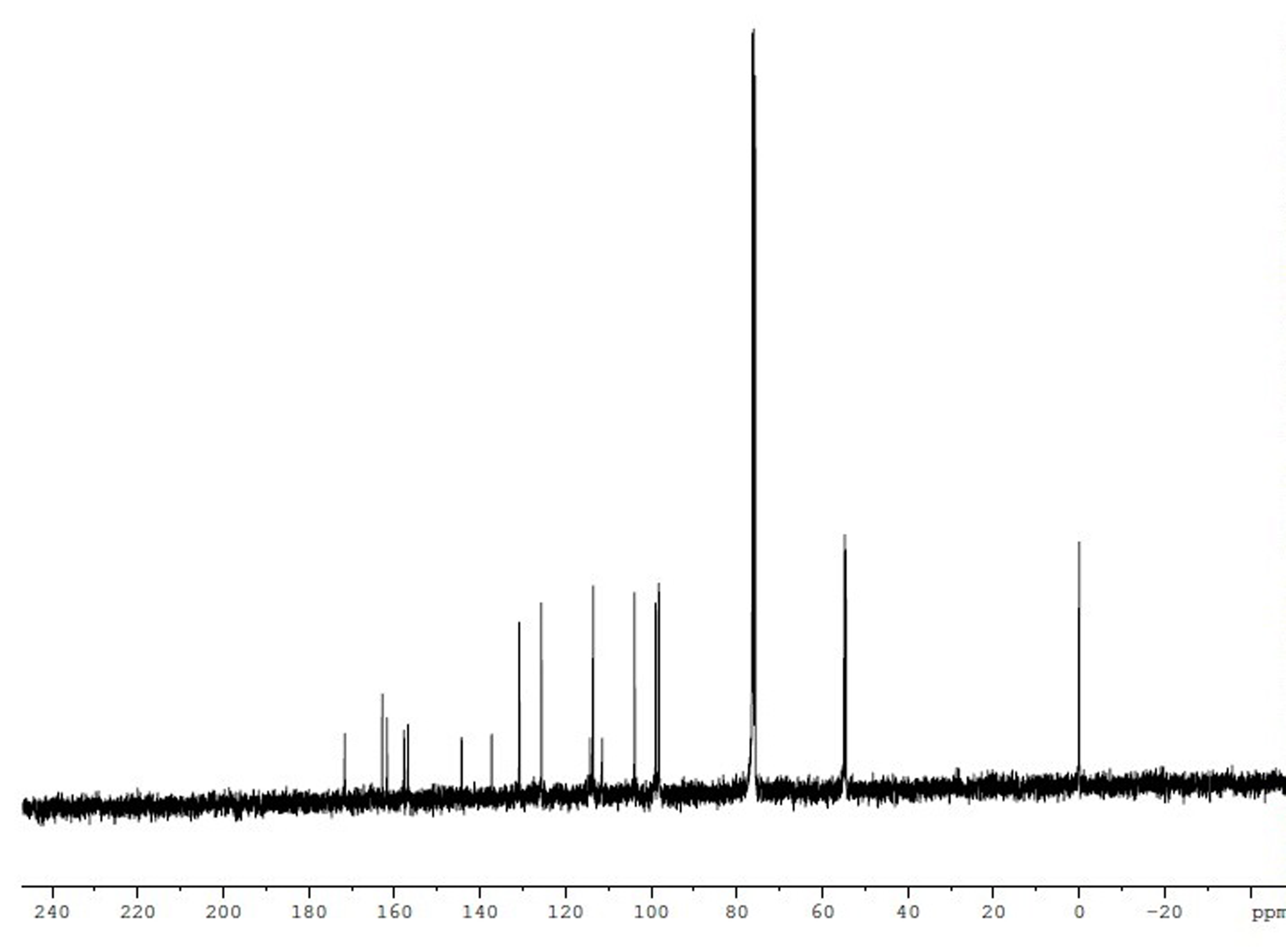


**Figure S_2_:** Carbon NMR profile of isolated compound from aerial parts of *L. nodiflora.*
